# The Impact of COVID-19 on Individuals with Hearing and Visual Disabilities during the First Pandemic Wave in Italy

**DOI:** 10.3390/ijerph181910208

**Published:** 2021-09-28

**Authors:** Luciano Bubbico, Saverio Bellizzi, Salvatore Ferlito, Antonino Maniaci, Raffaella Leone Guglielmotti, Giulio Antonelli, Giuseppe Mastrangelo, Luca Cegolon

**Affiliations:** 1 Department of Sensorineural Disabilities, INAPP/Italian Institute of Social Medicine, 00198 Rome, Italy; l.bubbico@inapp.org; 2 Medical Epidemiologist, Independent Consultant, 1200 Geneva, Switzerland; saverio.bellizzi@gmail.com; 3 Department of Surgical Medical Sciences and Advanced Technologies, University of Catania School of Medicine, 95124 Catania, Italy; ferlito@unict.it (S.F.); tnmaniaci29@gmail.com (A.M.); 4 Auximon Trainig Institute for Transactional Analysis, 00197 Rome, Italy; auxif@formazionepoiesis.it; 5 Biomedicine and Movement Sciences, Department of Neurosciences, University of Verona, 37129 Verona, Italy; Giulio.antonelli1991@gmail.com; 6 Department of Cardiac, Thoracic, Vascular Sciences & Public Health, Padua University, 35122 Padua, Italy; giuseppe.mastrangelo@unipd.it; 7 Public Health Department, Local Health Unit N.2 “Marca Trevigiana”, 31100 Treviso, Italy

**Keywords:** COVID-19, SARS-CoV-2, visual disability, hearing disability, lock-down, social restrictions, isolation, face masks

## Abstract

Background. The COVID-19 pandemic has imposed radical behavioral and social changes in the general population, significantly impacting the lives of individuals affected by disabilities. The aim of this study was to investigate the impact of COVID-19 on non-institutionalized subjects with sensorineural disabilities during the first COVID-19 wave in Italy. Methods. A 39-item online national survey was disseminated from 1 April 2020 to 31 June 2020 via social media throughout Italy to communities of individuals with proven severe sensorineural disabilities, affiliated to five national patient associations. The survey collected extensive information on the socio-demographic profile, health, everyday activities, and lifestyle of individuals with hearing and visual disabilities. Results. One hundred and sixty-three respondents with hearing (66.9%) and visual (33.1%) disabilities returned a usable questionnaire. The mean age of interviewees was 38.4 ± 20.2 years and 56.3% of them were females. Despite the vast majority of respondents (77.9%) perceiving their health status as unchanged (68.8% of interviewees with hearing deficits vs. 96.3% of those with visual impairments), about half the interviewees reported sleep disorders during lock-down, more likely those with visual deficits. Remote services were seemingly more effective for business than school activities. Furthermore, although just 18.8% of respondents rated remote rehabilitation care unsatisfactory, only 12.8% of interviewees felt supported by health and social services during the COVID-19 emergency. The vast majority of respondents were concerned about the future and the risk of SARS-CoV-2 contagion, particularly individuals with hearing impairments. Among the various risk mitigation measures, facemasks caused the greatest discomfort due to communication barriers, particularly among interviewees affected by hearing disabilities (92.2% vs. 45.7%). The most common request (46.5%) of respondents to reduce the inconveniences of the COVID-19 emergency country lock-down was improving the access to and delivery of health and social services for individuals with sensorineural disabilities (19.3%), followed by the use of transparent masks (17.5%). Conclusions. Although health protection measures such as face masks and social distancing play a key role in preventing and controlling the spread of SARS-CoV-2, the unmet needs of disabled individuals should be carefully considered, especially those affected by sensory disabilities. Tailored access to health and social services for individuals affected by sensorineural disabilities should be implemented. Additional actions should include the use of to face masks to reduce communication barriers linked to hearing-impairment, as well as the improvement of remote services, especially distance learning at school.

## 1. Background

In January 2020, the Chinese government announced the outbreak of COVID-19, a respiratory disease caused by a novel beta-coronavirus subsequently called SARS-CoV-2. On 11th March 2020, COVID-19 was declared a pandemic by the World Health Organization (WHO) [1,2], and a country lock-down was enforced in Italy. Risk mitigation measures against the spread of COVID-19 immediately focused on identifying, treating, and isolating active cases of the disease and introducing health protection measures such as social distancing, frequent hand washing, and use of face masks, even outdoors [3].

Social restrictions against the spread of SARS-CoV-2 inevitably impacted the everyday and professional lives of all individuals, compromising inter-personal physical contacts and communications between people [4]. The health impact of COVID-19, the fear of SARS-CoV-2 contagion, and the unpredictable trend of the epidemic generated a strong emotional influence in the general population worldwide. Psychological distress such as anxiety, depression, and post-traumatic stress disorders have already been experienced in populations hit by the H1N1 influenza, MERS-CoV, and Ebola epidemics [5,6].

According to the United Nations, the psychological impact of COVID-19 has been even worse than the physical consequence of the disease [7]. For instance, in the UK, patients suffering from mental health problems increased by around 50% during country lock-down [8]. Furthermore, sleep disorders emerged as one of the main health problems associated with the COVID-19 emergency, increasing by 57.1% in Italy during country lock-down [9]. Sleep disorders have already been identified as one of the most common health problems and an early health indicator of the general population [10].

Some subgroups, such as those affected by disabilities, are inevitably more vulnerable to COVID-19 and less resilient to the respective social restrictions. Disabled individuals in fact typically experience economic hardships, have lower educational levels, have lower employment rates, and experience more barriers in accessing health and social care services than the general healthy population do [11,12]. The risk of death for COVID-19 during the first two months of the pandemic in England and Wales was 11 times higher among individuals with disabilities [13]. If, on the one hand, disabilities associated with poverty may create precarious social conditions, making it difficult to observe physical distancing and personal hygiene [12], on the other hand, COVID-19 may also increase social isolation, psychological distress, and difficulties in the interpersonal relationships of disabled individuals, who have a higher demand of health and social care support. Indeed, a recent nationwide survey reported that the psychological distress associated with COVID-19 was significantly higher among subjects affected by pre-existing conditions or disabilities in Italy [14]. Disability affects 1 billion individuals worldwide [12], 5.2% of the Italian general population (over 3.1 million people) [15].

By interfering with their capacity of accessing health and social care, habilitation, and rehabilitation services, communication barriers can play a major role in the vulnerability of subjects affected by sensorineural disabilities. These difficulties may be amplified by the lack of accessible information on the pandemic, as not all governments have provided sign language interpretation during COVID-19 briefings [11].

In view of the above, as the impact of COVID-19 on individuals affected by sensorineural disabilities has been almost neglected thus far, we conducted a survey on disabled individuals affected by hearing and visual impairments during the first COVID-19 pandemic wave in Italy, with the goal of understanding the effect of social restrictions on these particular social categories and their unmet needs, thus assisting policy makers to design tailored public health policies [16].

## 2. Materials and Methods

This observational survey was conducted through a specific 39-item online questionnaire, disseminated in Italy from 1 April 2020 to 31 June 2020 via social media to communities of individuals with proven severe sensorineural disabilities and affiliated with five national patient associations. The online version of the questionnaire was created through the “Survey Administration App” on the Google Forms web platform and circulated through multiple web channels (Facebook and Instagram). Furthermore, the survey tool was also shared within the mailing lists of the main dedicated Italian patients’ associations: “Lega filo d’ oro” (“Golden thread league”), “Italian blind union,” “Italian families associated for the defense of the rights of the deaf people,” “Umbria Onlus,” “Let’s face deafness together,” and “Association of cochlear implant bearers” Onlus.

### 2.1. Inclusion Criteria

A convenience snow-balling sample was employed to recruit as many subjects with sensorineural disabilities as possible. Participation in the study was voluntary and there was no control on the number of study participants. All questionnaires returned were considered, but only subjects with hearing or visual impairments were included in this study.

Severe visual impairment is defined by the WHO [17] as a best corrected visual acuity of 3/60–6/60, whereas the term blindness implies full or almost full vision loss (corrected visual acuity <3/60).

Hearing impairment is in turn defined as a best hearing threshold acuity of 60–80 dB (severe hearing impairment); 80–95 dB (profound hearing loss); and >95 dB (complete hearing loss) [18].

By Italian law (Ministerial Decree N. 293/89 2017 and 148/92 2018), individuals with hearing disabilities are defined as those:

- suffering from pre-lingual deafness;
- with a mean sensorineural hearing deficit ≥60 dB for 500 Hz, 1000 Hz, and 2000 Hz frequency tones in the best ear.

According to the Italian Law (law 138 of 3 April 2001), subjects with visual disabilities are those with:

- residual visus < 3/10 in both eyes or in the best eye, even with refractory correction;
- binocular perimetric residue <30%.

### 2.2. The Questionnaire

The questionnaire used for the survey (Supplementary File S1) was designed by an interdisciplinary team composed by an otolaryngologist, a public health consultant, a psychologist, and a sociologist, with the support of patients’ associations. The same four experts and an ophthalmologist reviewed the survey instrument, contributing also to improve its quality and reducing the initial number of questions from 45 to 39. As the survey was conducted in an emergency situation, during the first COVID-19 pandemic wave, a pilot test was conducted only on 3 sensorineural disabled patients.

The questionnaire investigated the impact of lock-down and various health protection measures on various aspects of participants’ behavior, including health status, lifestyle, education, smart working, access to health and social care services, evaluation of remote rehabilitation services, use of personal protective equipment (PPE), and other risk mitigation measures.

The survey questionnaire was broken down into four sections:

- The first collected demographic data of study subjects: sex, age, type of disability, and geographical area of residence.
- In the second, the impact of the COVID-19 pandemic on education and working activities and on any changes in social relationships was delved into.
- The third dealt with aspects of health/social care, rehabilitation care, use of PPE, and their eventual side-effects.
- The last part of the questionnaire analyzed eventual repercussions of the COVID-19 pandemic on patients’ quality of life in terms of sleep disorders, concentration difficulties, and use of symptomatic drugs. Further free answers provided understanding and insights.

For each answer to items of Section 2, Section 3 and Section 4, a 4-point scale (unsatisfactory, fair, good, and excellent) was used.

Questionnaires not fully completed were ruled out.

This study was conducted in accordance with the Declaration of Helsinki. Ethical approval was obtained by the Italian National Institute for Deaf People. Informed consent was obtained from study participants before starting the survey online. Collection, storage, management, and analysis of survey data were conducted anonymously.

### 2.3. Lifestyle, Stress Management, and Related Stressors

In order to assess the impact of lock-down, risk and protective factors for psychological distress, including changes in routine activities and daily behaviors, were examined. Based on previous literature related to the COVID-19 pandemic [6,14,19,20], lifestyle and stress management items (e.g., 29-What were your daily life habits during lock-down?) and 6 stressors terms (e.g., 35-Have you experienced sleep disturbances during lock-down?; 36 Are you worried about the future?) were arranged.

Data analysis was performed using Stata 14.3 (Stata corporation, College station, Texas, USA). Descriptive statistics was performed, reporting frequencies and percentages. Univariable logistic analysis was also employed to contrast disability type (visual vs. hearing) by each explanatory factor. A *p*-value < 0.05 was considered statistically significant.

## 3. Results

After the exclusion of 25 questionnaires not fully completed, 163 study subjects with hearing and visual disabilities met the inclusion criteria.

The mean age of study participants (56.5% being females) was 38.4 ± 20.2 years. Sociodemographic and clinical features of respondents are summarized in Table 1. As can be seen, the vast majority of interviewees (86.9%) were living at least with one family member (partner, parent, or child) and 77.4% were living in a building with an open space (garden or terrace).

### 3.1. Lifehabits and Perceived Well-Being

As can be seen from Table 1, 85.9% (=122/163) of disabled interviewees had meals at regular time schedules during lock-down, and 44.8% (=73/163) reported weight gain. Moreover, whilst 75.5% of respondents were dedicated to recreational activities before the COVID-19 pandemic, this proportion reduced to 36.3% during lock-down (Table 2).

**Figure 1:**
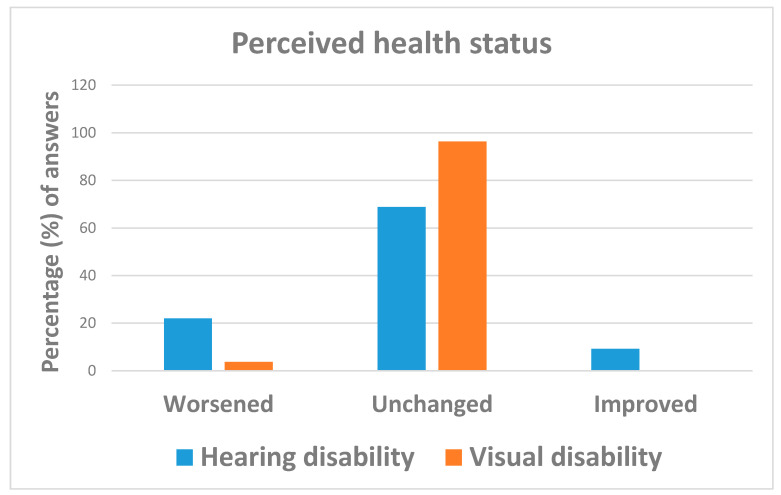
Answers to question: “How has your perceived health state changed as compared to before the COVID-19 emergency?”.

**Figure 2:**
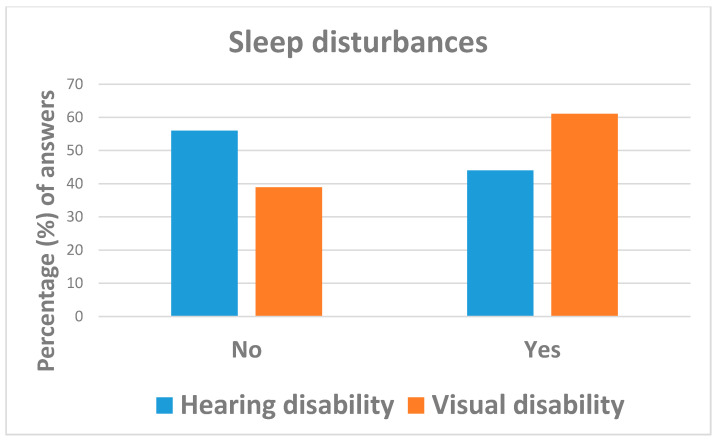
Answers to question: “Have you experienced sleep disturbances during lock-down?”.

Overall, the COVID-19 pandemic did not substantially change the everyday life of respondents, with 77.9% (=127/163) of them reporting a stable health status as compared to before the emergency, especially those affected by visual impairments (96.3% vs. 68.8%). Respondents with visual disabilities were less likely (OR = 0.12; 95%: 0.03; 0.53) to report a worsened health status during lock-down than those with hearing impairments (Table 2 and Figure 1). Despite 26.4% (=38/164) of subjects reporting regular sleeping schedules, about half of them (49.7% = 81/163) suffered from sleep disturbances, more likely those affected by visual disabilities (OR = 2.00; 95%CI: 1.03; 3.88) (Table 2 and Figure 2).

Albeit prolonged forced cohabitation during lock-down, for 65.5% (=95/116) of subjects, the respective household relationships did not vary as compared to before the COVID-19 emergency (Table 2). Moreover, only 10.6% (=17/160) were not able to keep in contact with friends/relatives during lock-down, more likely those affected by visual deficits (OR = 4.38; 95%CI: 1.42; 13.54), and 38.5% (=61/160) of interviewees reported improved contacts.

### 3.2. Education

**Figure 3:**
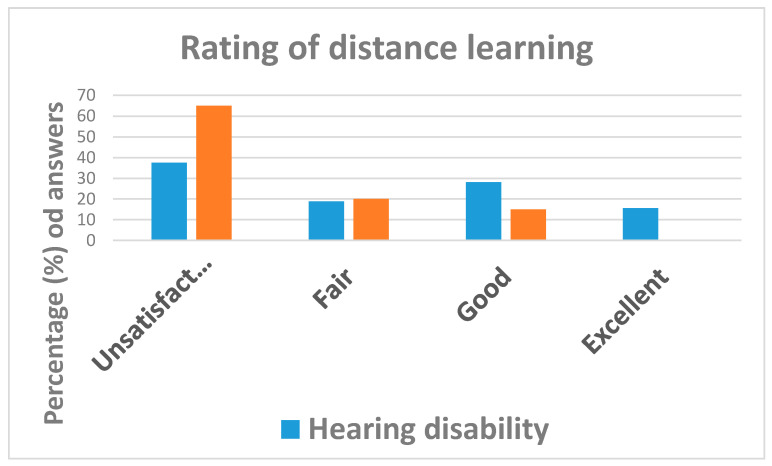
Answers to question: “How do you rate distance-learning?”.

As can be seen from Table 3, school activities continued during lock-down for 84.6% (=33/39) of student respondents. However, distance learning was rated unsatisfactory by 43.6% (=17/39) of students, of whom 65.0% were individuals affected by visual disabilities and 37.5% by hearing impairments (Figure 3).

**Figure 4:**
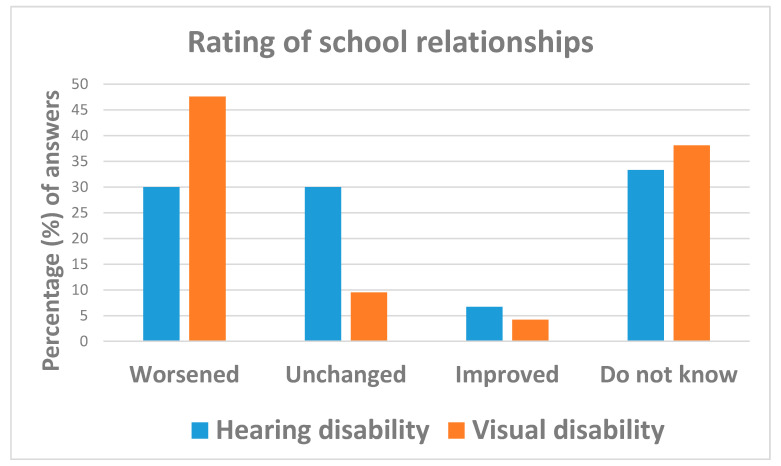
Answers to question: “How do you rate your current school relationships as compared to before the COVID-19 emergency?”.

A support teacher, available to 68.4% (=26/38) of students before the pandemic, was no longer available to 61.5% (=16/26) of them during country lock-down, particularly to those affected by visual disabilities. Social relationships with classmates were also significantly compromised by the COVID-19 emergency, worsening for 37.3% (=19/39) of interviewees as compared to before the emergency (Table 3 and Figure 4).

### 3.3. Work

**Figure 5:**
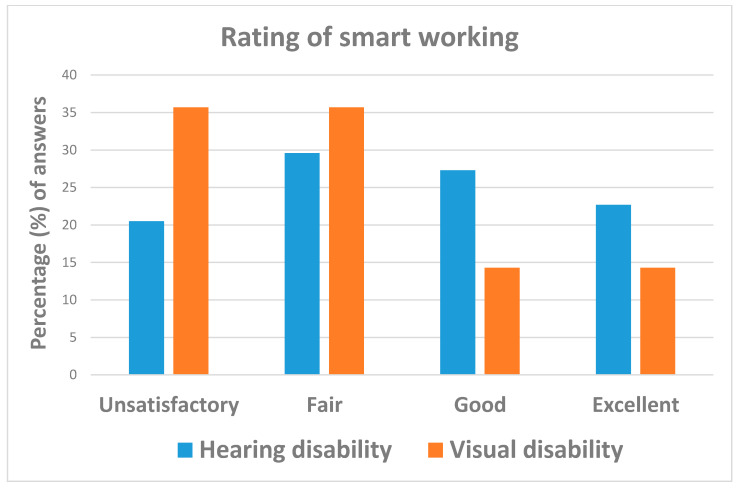
Answers to question: “How do you rate remote working?”.

The majority of workers (84.9% = 62/73) continued their job during the COVID-19 emergency, with 66.7% (=46/79) of them being equipped with remote services by the respective employer. As can be seen from Table 3 and Figure 5, although the relative estimates were not statistically significant, individuals affected by visual disabilities appreciated remote working more than those with hearing impairments.

**Figure 6:**
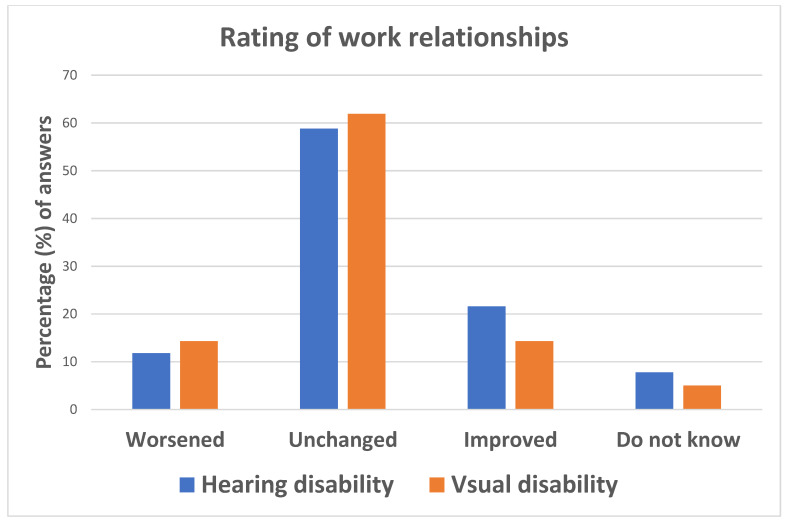
Answers to question: “How do you rate your current work relationships as compared to before the COVID-19 emergency?”.

The rating of work relationships was similar between those affected by visual and hearing impairments, with most of both groups predominantly classifying their relationship with colleagues as unchanged as compared to before the COVID-19 emergency (Figure 6).

### 3.4. Health, Social and Rehabilitation Services

**Figure 7:**
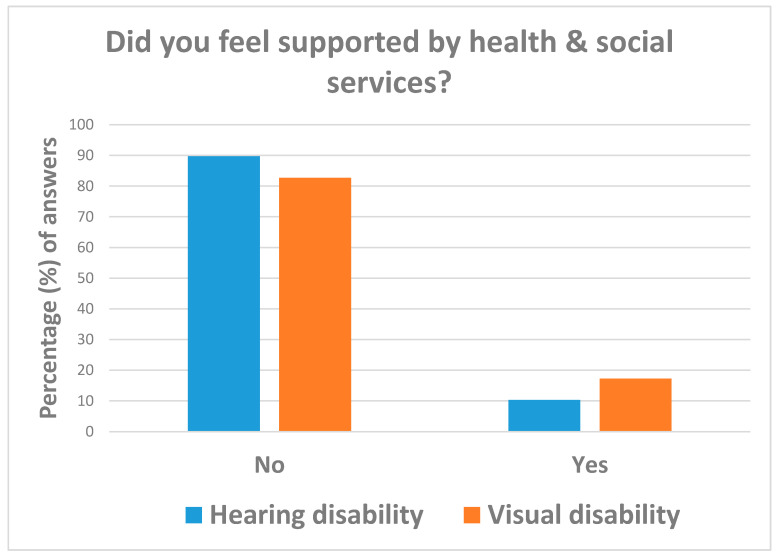
Answers to question: “Did you feel supported by health and social care services during the COVID-19 emergency?”.

**Figure 8:**
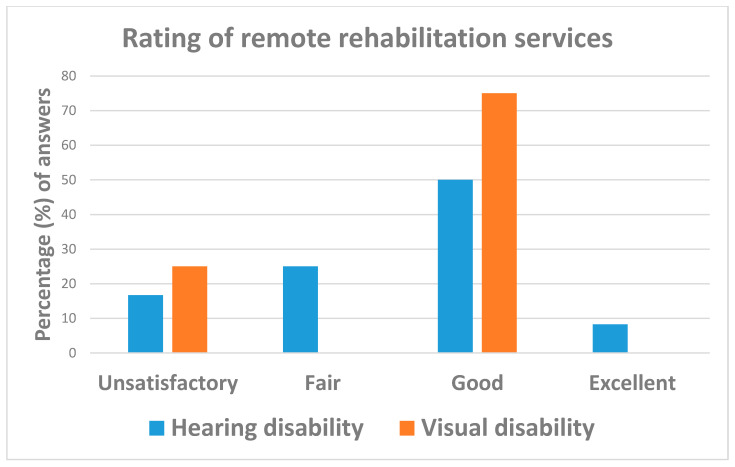
Answers to question: “How do you rate remote rehabilitation services?”.

As can be seen from Table 4, most respondents (87.3% = 130/163) reported unsatisfactory support from health and social care services during the COVID-19 emergency, with balanced figures by disability type (Figure 7). By contrast, 56.3% (=9/16) of interviewees considered remote rehabilitation services as good (75.0% for disabled with visual deficits vs. 50.0% of those with hearing impairments) (Table 4 and Figure 8). Respondents following rehabilitation programs before the COVID-19 emergency were 32.5% (=38/117) more likely those affected by visual disabilities (OR = 3.06; 95%CI: 1.37; 6.89). Due to the COVID-19 emergency, rehabilitation services had to be suspended for 60.5% (=23/38) of interviewees, with individuals affected by visual impairments less likely to continue their rehabilitation programs (OR = 0.12; 95%CI: 0.02; 0.68) than those with hearing disabilities and 79.0% (=15/19) of respondents continuing their rehabilitation programs by distance (Table 4).

### 3.5. Emotional Aspects

**Figure 9:**
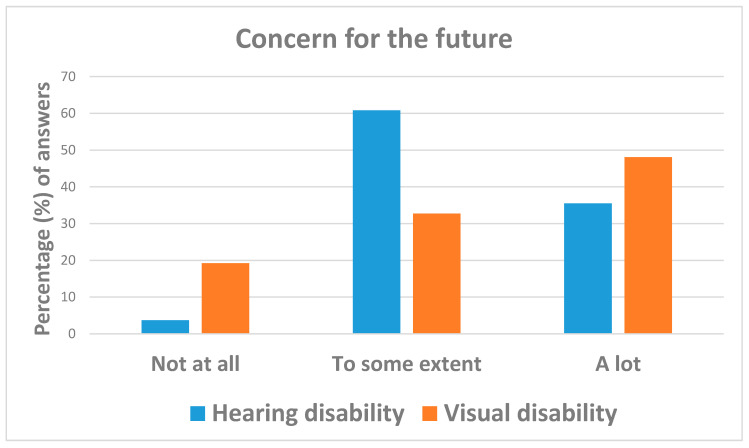
Answers to question: “*Are you worried about the future?*”.

**Figure 10:**
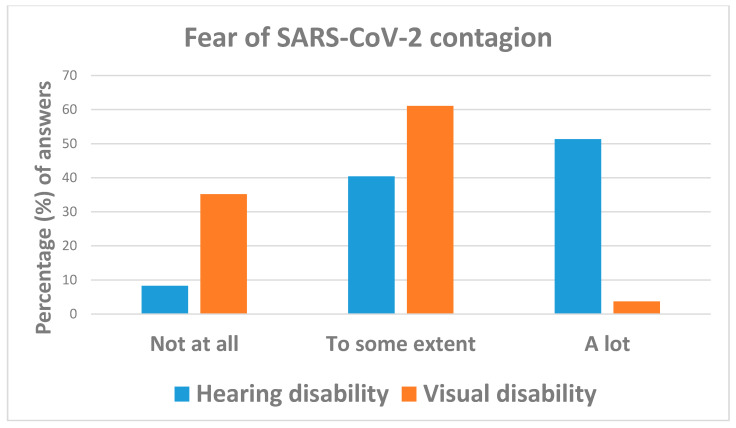
Answers to question: “Are you worried about SARS-CoV-2 contagion and hospitalization?”.

Table 4 show that concern about the future (Figure 9) and fear of SARS-CoV-2 contagion (Figure 10) were remarkably higher among interviewees with hearing impairments (51.4%) than those with visual disabilities (3.7%). Specifically, individuals with visual deficits were less likely to be concerned “a lot” (OR = 0.02; 95%CI: 0.00; 0.08) or “to some extent” (OR = 0.36; 95%CI: 0.14; 0.88) than those with hearing impairments. Likewise, respondents with visual disabilities were less likely to be worried “a lot” (OR = 0.26; 95%CI: 0.07; 0.93) or “to some extent” (OR = 0.10; 95%CI: 0.03; 0.37) about the future than those with hearing problems.

**Figure 11:**
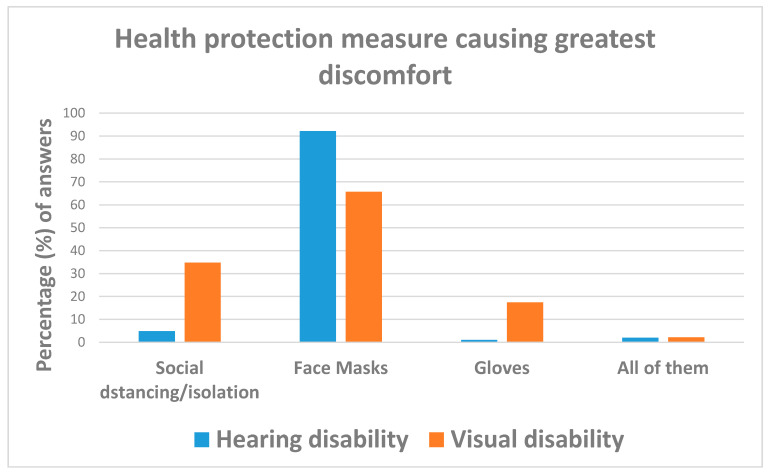
Answers to question: “Which health protection measure caused the greatest discomfort to you during the COVID-19 emergency?”.

Facemasks caused the greatest discomfort (77.7% = 115/148), followed by isolation/social distancing (14.1% = 21/148). However, whilst isolation and social distancing caused more inconvenience in individuals with visual disabilities (34.9% vs. 4.9%), facemasks were predominantly a discomfort for respondents with hearing impairments (92.2% vs. 45.7%). Visually impaired respondents were less likely (OR = 0.07; 95%CI: 0.02; 0.21) to express discomfort for the use of face masks than those with hearing deficits (Table 4 and Figure 11).

Finally, improved health and social care (19.3% = 2/114) and the use of transparent masks (17.5% = 20/114) were the most suggested solutions to reduce the burden of risk mitigation measures against the spread of SARS-CoV-2. Individuals affected by visual disabilities were less likely to propose transparent masks (OR = 0.09; 95%CI: 0.02; 0.50) than those with hearing impairments (Table 4).

## 4. Discussion

This online survey investigated the impact of health protection measures against the spread of SARS-CoV-2 on a sample of individuals affected by sensorineural disabilities during the first COVID-19 pandemic wave in Italy. Social media proved to be a valuable, quick, and low-cost instrument to distribute the survey in a relatively short time. However, access to social media may be hampered for some disabled individuals, who therefore may have been excluded from participating in this study. Despite a relatively limited statistical power and low representativeness of the sample, the findings of this survey are still useful to obtain indications of the health and psychological stress suffered by individuals affected by hearing and visual disabilities during the COVID-19 emergency in Italy.

In particular, the pandemic seemingly had a negative influence on the everyday life of disabled individuals affected by sensorineural impairments. Among the most relevant issues experienced by study subjects were a fear of SARS-CoV-2 contagion and concern about the future, especially among individuals affected by hearing deficits, who were also more likely to be worried about communication barriers caused by the mandatory use of face masks. Furthermore, most interviewees expressed dissatisfaction about the access/delivery of health and social care services during the emergency. Whilst remote services were relatively appreciated for business activities and rehabilitation programs, distance learning and relationships with schoolmates were instead rated unsatisfactory.

Both disabled groups expressed dissatisfaction with the access and delivery to health/social care services, including accident and emergency, and the probability for anguish linked to possible communication barriers due to face masks when dealing with healthcare staff. This may generate concern and fear of abandonment of not being able to have a caregiver available, an indispensable figure given their low sense of autonomy. This bad feeling could play a critical role in developing future psychological distress among the most vulnerable individuals.

As recently recommended by the WHO, in medical care facilities “*when caring for members of different populations, including deaf communities,*” the use of face masks introduces potential harms and risks that should be carefully considered [21]. It is therefore recommended to design patient-centered health care protocols tailored at the clinical needs of all users, with a reception phase capable of promptly understanding any discomfort experienced by disabled individuals in the first place [18,21].

Specific software technological applications are now available for the conversion of texts into voice and vice-versa, allowing the provision of an adequate level of communication for patients affected by hearing disabilities, thus maximizing their compliance and resilience with health care [22]. Unfortunately, resources for these advanced services are limited, causing potential frustration in patients and health/social care workers.

In the present survey, half respondents suffered from sleep disorders (falling asleep, sleep maintenance, and daytime sleepiness), more likely those affected by visual disabilities. Sleep disorders significantly increased during lock-down [23], particularly among people with visual impairments, probably for a reduced perception of light, which plays a fundamental role in the circadian rhythm [24]. Recent studies reported that the prevalence of insomnia and hypersomnia is around 40% and continuously increasing among individuals fearing SARS-CoV-2 contagion and social isolation [25]. Considering the enormous impact sleep disorders have on the global population, governments should consider setting up ad hoc programs to assist patients in overcoming sleep disturbances. Cognitive behavioral therapy (CBT) may support individuals with insomnia by modifying their behavior in terms of stimuli control, sleep restriction, sleep hygiene, relaxation training and cognitive therapy [26]. CBT can be delivered in groups, single face-to-face sessions, or online [27].

Despite the limits of distance learning, school support was still guaranteed to the majority of respondents in the present study. Such remote services represent one of the key elements of social integration for individuals affected by disabilities. However, whilst remote services somehow ensured the continuation of business activities and rehabilitation programs by distance during the COVID-19 emergency in Italy, modern technologies were not as effective to maintain school activities and the respective interpersonal relationships for disabled students. The efficacy of distance learning is in fact influenced by multiple methodological aspects [28,29].

From the analysis of free open questions, better access to health and social care and the use of transparent masks were the most frequent solutions proposed to mitigate the untoward impact of COVID-19 social restrictions. Whilst more effective against the spread of droplets, allowing also to maintain the physiological temperatures of upper airways under cold environmental conditions [30], traditional face masks can in fact cause communication barriers, especially in individuals with hearing disabilities, typically relying on lip reading and facial expressions. Nonetheless, transparent masks are not a certified PPE yet; hence, they are not accessible everywhere. Alternatively, protective face shields, which are classified by the US Food and Drug Administration as class 1 (low risk) medical devices based upon the level of risk for a user or a patient, could contribute to mitigate some of the communication barriers and anxiety experienced by individuals affected by hearing impairments [31,32].

Virtual tele-consultations could also be a potential solution for some disabled patients, and real-time subtitles are offered for free from some platforms (Google Meet and Microsoft Teams). Digital healthcare received a big boost during the COVID-19 emergency, accelerating the implementation of telemedicine services [11]. Tele-audiology options are already common practice in some countries, allowing the remote transmission of images by smartphone or tablets and the examination of patients through an online health platform. Tele-audiology solutions are also useful for the distance training of health professionals involved in the management of deafness and hearing loss [33].

## 5. Conclusions

In agreement with recent reports, this study confirmed that the prevalence of psychological distress during the first COVID-19 pandemic wave—in terms of the fear of SARS-CoV-2 contagion and concern for the future—was high among individuals affected by sensorineural disabilities in Italy [22].

Isolation and concerns for the future may have negative impacts on the psychological wellbeing of people with hearing and visual deficits, who already face stressors in the course of their lives as compared to the general population [34].

Remote services were more appreciated for business than school activities.

The use of face masks may generate significant communication barriers for sensory disabled individuals, particularly in those affected by hearing deficits. The use of class 1 PPE face shields could be introduced in health facilities and schools to mitigate the communication discomfort caused by face masks.

The COVID-19 pandemic has put health services under pressure, especially those delivering social and psychological care [35], spontaneously accelerating the evolution of novel technology and implementing remote services such as telemedicine, tele-rehabilitation, tele-consultation, and new digital infrastructures for modern data communication [11].

Future studies investigating the knowledge of novel tele-medicine technologies and their applications among patients with neurosensory disabilities are recommended, assessing the awareness of new paths of health and social care, with the aim of improving the access and delivery of health/social services in these particular social categories, reducing patients’ waiting lists, and containing the cost of care.

## Figures and Tables

**Table 1:** Distribution of sociodemographic factors by disability type (visual vs. hearing). Number, column percentage (%), and odds ratio (OR) with 95% confidence interval (95%CI). M = Missing information; N.S. = Nonsignificant.

VARIABLES	STRATA	Total (*n* = 164) (Col %)	DISABILITY TYPE		Univariable Logistic Analysis	
			Hearing *n* = 109 (66.9%)	Visual *n* = 54 (33.1%)	OR (95%CI)	*p* Value
**Sociodemographic Factors**						
Sex (Missing: 3)	Female	90 (56.3)	67 (62.6)	23 (43.4)	reference	
	Male	70 (43.7)	40 (37.4)	30 (56.6)	2.18 (1.12; 4.27)	0.020
Age (years) 38.4 ± 20.2	<23	41 (25.2)	25 (22.9)	16 (29.6)	reference	
	23–38	40 (24.5)	29 (26.6)	11 (20.4)	0.59 (0.23; 1.51)	N.S.
	39–53	38 (23.3)	23 (21.1)	15 (27.8)	1.02 (0.41; 2.51)	N.S.
	54+	44 (26.9)	32 (29.4)	12 (22.2)	0.59 (0.24; 1.46)	N.S.
Geographic area (Missing: 1)	Northern Italy	97 (59.9)	62 (56.9)	35 (66.0)	reference	
	Central Italy	46 (28.4)	35 (32.1)	11 (20.8)	0.56 (0.25; 1.23)	N.S.
	Southern Italy	19 (11.7)	12 (11.0)	7 (13.2)	1.03 (0.37; 2.07)	N.S.
Occupational status (Missing: 5)	Unemployed	11 (6.9)	11 (10.4)	0	1	
	Retired	34 (21.4)	20 (18.9)	14 (26.9)	1.12 (0.44; 2.87)	N.S.
	Student	39 (24.5)	24 (22.6)	15 (28.1)	reference	N.S.
	Worker	75 (47.2)	51 (48.1)	23 (44.2)	0.72 (0.32; 1.62)	N.S.
Who do you live with? (Missing: 18)	At least with one family member	126 (86.9)	86 (88.7)	40 (83.3)	reference	
	Alone	17 (11.7)	11 (11.3)	6 (12.5)	1.17 (0.41; 3.40)	N.S.
	in a care home	2 (1.4)	0	2 (4.2)	1	N.S.
External open space available in your place (M: 18)	No	33 (22.6)	26 (26.8)	7 (14.6)	reference	
	Yes	113 (77.4)	71 (73.2)	41 (85.4)	2.14 (0.86; 5.38)	N.S.

**Table 2:** Distribution of health status and lifestyle factors by disability type (visual vs. hearing). Number, column percentage (%), and odds ratio (OR) with 95% confidence interval (95%CI). M = Missing information; N.S. = Nonsignificant.

VARIABLES	STRATA	Total (*n* = 164) (Col %)	DISABILITY TYPE		Univariable Logistic Analysis	
			Hearing *n* = 109 (66.9%)	Visual *n* = 54 (33.1%)	OR (95%CI)	*p* Value
Health status and Lifestyle Habits						
Recreational activities before the COVID-19 emergency (M: 20)	No	35 (24.5)	28 (29.2)	7 (14.9)	reference	
	Yes	108 (75.5)	68 (70.0)	40 (85.1)	2.35 (0.94; 5.87)	N.S.
Recreational activities during the COVID-19 emergency (M: 39)	No	79 (63.7)	54 (67.5)	25 (56.8)	reference	
	Yes	45 (36.3)	26 (32.5)	19 (43.2)	1.58 (0.74; 3.37)	N.S.
Everyday habits during lock down (M: 23)	I always dress as I should go out	103 (73.6)	69 (71.9)	34 (77.3)	reference	
	I always wear pajamas all day	37 (26.4)	27 (28.2)	10 (22.7)	0.75 (0.33; 1.73)	N.S.
Sleeping habits during lock down (M: 19)	Regular schedule	106 (73.6)	68 (70.0)	38 (79.2)	reference	
	Whenever I wish	38 (26.4)	28 (29.2)	10 (20.8)	0.64 (0.28; 1.46)	N.S.
Sleep disorders during lock down (M: 2)	No	82 (50.3)	61 (56.0)	21 (38.9)	reference	
	Yes	81 (49.7)	48 (44.0)	33 (61.1)	2.00 (1.03; 3.88)	0.042
Eating habits during lock down (M: 21)	Regular schedule	122 (85.9)	81 (85.3)	41 (87.2)	reference	
	Whenever I wish	20 (14.1)	14 (14.7)	6 (12.8)	0.85 (0.30; 2.37)	N.S.
BMI (kg/m^2^) (M: 9)	<25	106 (68.8)	72 (71.3)	34 (64.2)	reference	
	25+	48 (31.2)	29 (28.7)	19 (35.1)	1.38 (0.68; 2.82)	N.S.
Increase in body weight during lock down	No	90 (55.2)	61 (56.0)	29 (53.7)	reference	
	Yes	73 (44.8)	48 (44.0)	25 (46.3)	1.10 (0.57; 2.11)	N.S.
How has your perceived health state changed as compared to before the COVID-19 emergency?	Improved	10 (6.1)	10 (9.2)	0	1	
	Unchanged	127 (77.9)	75 (68.8)	52 (96.3)	reference	N.S.
	Worsened	26 (16.0)	24 (22.0)	2 (3.7)	0.12 (0.03; 0.53)	0.005
Keeping in contact with friends/family through remote modern technology during lock-down? (M:3)	More than before	61 (38.1)	46 (42.2)	15 (29.4)	reference	
	As before	82 (51.3)	56 (51.4)	26 (51.0)	1.42 (0.68; 3.00)	N.S.
	Less than before	17 (10.6)	7 (6.4)	10 (19.6)	4.38 (1.42; 13.54)	0.010
How do you rate your family relationships during lock-down? (M: 18)	Improved	29 (20.0)	21 (21.7)	8 (16.7)	reference	
	Unchanged	95 (65.5)	66 (68.0)	29 (60.4)	1.14 (0.45; 2.86)	N.S.
	Worsened	11 (7.6)	5 (5.2)	6 (12.5)	3.15 (0.75; 13.29)	N.S.
	Do not know	10 (6.9)	5 (5.2)	5 (10.4)	2.63 (0.60; 11.57)	N.S.

**Table 3:** Distribution of answers by disability type (visual vs. hearing). Number, column percentage (%), and odds ratio (OR) with 95% confidence interval (95%CI). M = Missing information; N.S. = Nonsignificant.

VARIABLES	STRATA	Total (*n* = 164) (Col %)	DISABILITY TYPE		Univariable Logistic Analysis	
			Hearing *n* = 109 (66.9%)	Visual *n* = 54 (33.1%)	OR (95%CI)	*p* Value
**Analysis restricted to students (*n* = 39)**						
School activities ongoing during the COVID-19 emergency	No	6 (15.4)	3 (12.5)	3 (20.0)	reference	
	Yes	33 (84.6)	21 (87.5)	12 (80.0)	1.49 (0.59; 3.72)	N.S.
Support teacher available before the COVID-19 emergency (M: 1)	No	12 (31.6)	11 (47.8)	1 (6.7)	reference	
	Yes	26 (68.4)	12 (52.2)	1 (93.3)	7.37 (1.43; 38.08)	0.022
Support teacher interrupted during the COVID-19 emergency (M: 1)	No	15 (48.4)	8 (53.3)	7 (43.8)	reference	
	Yes	16 (52.6)	7 (46.7)	9 (56.3)	1.47 (0.36; 6.05)	N.S.
How do you consider	Excellent	5 (12.8)	5 (15.6)	0	1	
distance learning for	Good	8 (20.5)	9 (28.1)	3 (15.0)	0.31 (0.07; 1.41)	N.S.
your disability?	Fair	9 (23.1)	6 (18.8)	4 (20.0)	0.62 (0.14; 2.73)	N.S.
	Unsatisfactory	17 (43.6)	12 (37.5)	13 (65.0)	reference	
How do you rate your current school relationships as compared to before the COVID-19 emergency?	Improved	3 (5.9)	2 (6.7)	1 (4.2)	reference	
	Unchanged	11 (21.6)	9 (30.0)	2 (9.5)	0.44 (0.03; 7.67)	N.S.
	Worsened	19 (37.3)	9 (30.0)	10 (47.6)	2.22 (0.17; 28.86)	N.S.
	Do not Know	18 (35.3)	10 (33.3)	8 (38.1)	1.60 (0.12; 20.99)	N.S.
**Analysis restricted to workers (*n*** **=** **74)**						
Have you continued your job during the COVID-19 emergency? (M: 2)	No	11 (15.3)	6 (11.8)	5 (23.8)	reference	
	Yes	61 (84.7)	45 (88.2)	16 (76.2)	0.42 (0.11; 1.59)	N.S.
Remote-working facilitated by the employer duringthe COVID-19 emergency (M: 2)	No	23 (33.3)	17 (33.3)	7 (33.3)	reference	
	Yes	46 (66.7)	32 (66.7)	14 (66.7)	1.00 (0.34; 2.97)	N.S.
How do you rate remote working? (M: 18)	Excellent	13 (23.3)	8 (19.1)	5 (35.7)	reference	
	Good	18 (32.1)	13 (31.0)	5 (35.7)	0.62 (0.13; 2.82)	N.S.
	Fair	14 (25.0)	12 (28.6)	2 (14.3)	0.27 (0.04; 1.73)	N.S.
	Unsatisfactory	11 (19.6)	9 (22.4)	2 (14.3)	0.36 (0.05; 2.37)	N.S.
How do you rate your relationships with colleagues as compared to before the COVID-19 emergency? (M: 5)	Improved	9 (12.5)	6 (11.8)	3 (14.3)	reference	
	Unchanged	43 (59.7)	30 (58.8)	13 (61.9)	0.87 (0.19; 4.01)	N.S.
	Worsened	14 (19.4)	11 (21.6)	3 (14.3)	0.55 (0.08; 3.59)	N.S.
	Not answered	6 (8.3)	4 (7.8)	2 (5.00)	1.00 (0.11; 8.95)	N.S.

**Table 4:** Distribution of answers by disability type (visual vs. hearing). Number, column percentage (%), and odds ratio (OR) with 95% confidence interval (95%CI). M = Missing information.

VARIABLES	STRATA	Total (*n* = 164) (Col %)	DISABILITY TYPE		Univariable Logistic Analysis	
			Hearing *n* = 109 (66.9%)	Visual *n* = 54 (33.1%)	OR (95%CI)	*p* Value
**Access to health and social care services**						
Did you feel supported by health & social services during the COVID-19 emergency? (M: 14)	No	130 (87.3)	87 (89.7)	43 (82.7)	reference	
	Yes	19 (12.8)	10 (10.3)	9 (17.3)	1.82 (0.69; 4.81)	N.S
Rehabilitation programs before the COVID-19 emergency (M: 46)	No	79 (67.5)	58 (7.3)	21 (51.2)	reference	
	Yes	38 (32.5)	18 (23.7)	20 (48.8)	3.06 (1.37; 6.89)	0.007
Rehabilitation programs during the COVID-19 emergency (M: 82)	No	23 (60.5)	9 (50.0)	14 (70.0)	reference	
	Yes	15 (39.5)	9 (50.0)	6 (30.0)	0.12 (0.02; 0.68)	0.017
Remote rehabilitation during COVID-19 emergency	No	4 (21.1)	1 (25.0)	3 (75.0)	reference	
	Yes	15 (79.0)	11 (73.3)	4 (67.7)	0.35 (0.02; 1.37)	N.S.
If you are following remote rehabilitation during the COVID-19 emergency, how do you rate these distance services?	Excellent	1 (6.3)	1 (8.3)	0	NA	N.S.
	Good	9 (56.3)	6 (50.0)	3 (75.0)	reference	
	Fair	3 (18.8)	3 (25.0)	0	NA	N.S.
	Unsatisfactory	3 (18.8)	2 (16.7)	1 (25.0)	1.41 (0.06; 15.99)	N.S.
**Emotional impact of COVID-19 pandemic**						
Are you worried about the risk of SARS-CoV-2 contagion and hospitalization? (M: 2)	Not at all	28 (17.2)	9 (8.3)	19 (35.2)	reference	
	To some extent	77 (47.2)	44 (40.4)	33 (61.1)	0.36 (0.14; 0.88)	0.026
	A lot	58 (35.6)	56 (51.4)	2 (3.7)	0.02 (0.00; 0.08)	<0.001
Are you worried about the future? (M: 4)	Not at all	14 (8.8)	4 (3.7)	10 (19.2)	reference	
	To some extent	82 (51.6)	65 (60.8)	17 (32.7)	0.10 (0.03; 0.37)	0.001
	A lot	63 (39.6)	38 (35.5)	25 (48.1)	0.26 (0.07; 0.93)	0.039
Which health protection measure caused the greatest discomfort to you? (Total free answers: 149) (M: 15)	Social distancing/isolation	21 (14.1)	5 (4.9)	16 (34.8)	reference	
	Face masks	115 (77.7)	94 (92.2)	21 (45.7)	0.07 (0.02; 0.21)	<0.001
	Gloves	9 (6.1)	1 (1.0)	8 (17.4)	2.50 (025; 25.15)	N.S.
	All of them	3 (2.0)	2 (2.0)	1 (2.2)	0.16 (0.01; 2.11)	N.S.
Solutions proposed to improve quality of life of sensorineural disabled in the COVID-19 emergency (Total free answers: 114) (M: 49)	Improving access/delivery of health care and social services to disabled	22 (19.3)	10 (13.0)	12 (32.4)	reference	
	Use of transparent masks	20 (17.5)	18 (23.4)	2 (5.4)	0.09 (0.02; 0.50)	0.006
	Mitigating social restrictions	21 (18.4)	13 (16.9)	8 (21.6)	0.51 (0.15; 1.73)	N.S.
	Better information/communications on COVID-19	19 (16.7)	13 (16.9)	6 (16.2)	0.38 (0.11; 1.38)	N.S.
	Better security control (*n* = 6)	20 (17.5)	15 (19.5)	5 (13.5)	0.28 (0.07; 1.03)	0.056
	More awareness on disabilities (*n* = 8)					
	Other (*n* = 6)					
	Do not know	12 (10.5)	8 (10.4)	4 (10.8)	0.42 (0.10; 1.80)	N.S.

## Data Availability

Data generated and analysed for this study are not publicly available, since they were purposively collected by the authors for the present study, but are available from the corresponding author on reasonable request.

## Supplementary Materials

The following are available online at https://www.mdpi.com/article/10.3390/ijerph181910208/s1, Supplementary File S1: A survey on the impact of COVID-19 on individuals with hearing and visual disability during the first pandemic wave in in Italy.
