# Supplementary material for: The Impact of COVID-19 on Individuals with Hearing and Visual Disabilities during the First Pandemic Wave in Italy
Source: Int J Environ Res Public Health. 2021 Sep 28;18(19):10208. doi: 10.3390/ijerph181910208 (PMC8508015; doi:10.3390/ijerph181910208)
Supplement: Supplementary file 1 [file ijerph-18-10208-s001.zip › ijerph-1364387-supplementary.pdf]

INAPP
ISTITUTO NAZIONALE PER L'ANALISI DELLE POLITICHE PUBBLICHE

Neurosensorial Research

A survey on the impact of COVID-19 on individuals with hearing and visual disabilty during the first pandemic wave in in Italy

Research Group for Neurosensorial Disability

This survey is part of a large study on social inclusion of individuals with hearing and/or visual disabilities. This study has been conducted by a multidisciplinary research group (Research group of neuro-sensorial disability, chiefed by Dr. Luciano Bubbico) of the National Institute of Analysis of Public Policies INAPP.

This survey aims to provide a quick overview of the impact of COVID-19 pandemic experienced by individuals with hearing and/or visual disability, with the view of proposing potential interventions to improve their quality of life.

We kindly invite you to quickly answer the questions. The response time allowed is a few minutes. we apologies if the questionnaire is not readable from a screen reader per blind people, if the language used is difficult to understand and cannot be translated in sign language.

The questionnaire is anonymous and the sensitive data will treated in compliance with the current Italian privacy law.

Thank you in advance for your cooperation, your answers will be extremely useful to address emerging issues.

SECTION 1
(SOCIO-DEMOGRAPHIC INFORMATION)

1. Height and weight ………………………………………………………………………….

2. Gender
- o M
- o F

3. Educational level
- o Nursery
- o Primary school
- o Junior secondary school
- o Secondary school
- o University
- o Other (specify …………………………………………………………………………….)

4. What region do you live in now?
- o Valle d'Aosta
- o Piedmont
- o Lombardy
- o Liguria
- o Veneto
- o Emilia Romagna
- o Trentino Alto Adige
- o Friuli Venzia Giulia
- o Tuscany
- o Lazio
- o Umbria
- o Marche
- o Campania
- o Abruzzo
- o Molise
- o Basilicata
- o Calabria
- o Apulia
- o Sicily
- o Sardinia

5. Occupational status
- o Student
- o Worker (specify ....)

- Unemployed
- Retired
- Other…………………

6. What kind of disability do you have?

- hearig
- visual
- Other (specify …….........................)

7. Do you have any other deficit associated with your disability?

- Yes
- No

if so, which ones?

- Visual
- Hearing
- neurological
- cognitive
- engines
- cardiological
- I do not know
- other (specify ……………………………………………………………………….)

8. Does your disability need support by a carer for everyday life activites?

- Yes
- No

If so, was the above support interrupted due to the COVID-19 emergency?

- Yes
- No

9. Is the municipality where you currently live offering you social services?

- Yes
- No

if so, which ones? ………………………………………………………………………………………..

SECTION 2a

(FOR STUDENTS)

10. If you are a student, are you continuing your school activities during the COVID-19 emergency?

- Yes
- No

If so, what learning mode are you following?

- Distance learning using live platforms

- Lessons on platforms dedicated for your specific disability
- I study on my own
- Tutored personalized study
- Other (specify)

11. How do you rate distance learning?

- Unsatisfactory
- Fair
- Good
- Excellent

12. If you are a student, were you benefiting from a support teacher before the COVID-19 emergency?

- Yes
- No

13. If you are a student, were you using any other school support before the COVID-19 emergency?

- Yes
- No

If so, specify ....

14. Was the above learning support interrupted due to the COVID-19 emergency?

- Yes
- No

15. How do you rate your current school relationships as compared to before the COVID-19 emergency?

- Improved
- Worsened
- Unchanged
- I do not know

SECTION 2b
(FOR WORKERS)

16. If you are a worker, are you continuing your job during the COVID-19 emergency?

- Yes
- No

17. Do you benefit from special support from your employer?

- Yes

- No

If so, which one?

- Smart working
- Tele-working
- Other (specify ....)

If so, how do you rate remote working?

- Unsatisfactory
- Fair
- Good
- Excellent

18. How do you rate your current relationships with colleagues as compared to before the COVID-19 emergency?

- Improved
- Worsened
- Unchanged
- I do not know

19. Do you have all necessary equipment for your smart working (computer or other electronic device, internet connection, etc)?

- Yes
- No

SECTION 3

(HEALTH, REHABILITATION & SOCIAL CARE DURING THE COVID-19 EMERGENCY)

20. What kind of social welfare scheme is currently covering you?

- Civil disability
- Carer's allowance
- Other (specify ....)

21. Were you following rehabilitation programs before the COVID-19 emergency?

- Yes
- No

If so, can you please specify?

- Psychomotor skills
- Psychological
- Speech therapy
- Physiotherapy
- Neuropsychological functions empowering
- Other (specify …………………..…………………..…………………….….)

22. Are you continuing your rehabilitation during the COVID-19 emergency?

- Yes

- No

If so, are you following remote rehabilitation programs?

- Yes
- No

If so, how do you rate rehabilitation services by distance?

- Insufficient
- Sufficient
- Discrete
- Excellent

23. Did you feel supported by health and social care services during the COVID-19 emergency?

- Yes
- No

SECTION 4

(QUALITY OF LIFE DURING THE COVID-19 EMERGENCY)

24. Have you received clear information on prevention and control of SARS-CoV-2?

- Yes
- No

If so, where did you find them?

- Associations
- Television
- Internet
- Relatives and friends
- Other (specify …………………………………………….)

25. Who do you live with?

- Parents
- Partner
- Children
- Friends
- Other adult support figure
- Alone
- Other (specify …………………………………………………....)

26. Are external open spaces (garden, terrace, shared spaces) available in your place?

- Yes
- No

27. Who provides you with basic needs (food, medicines, etc.)?

- Myself
- One or more of my housemates
- Voluntary associations
- Municipal social care service
- Other (specify…………………..............................)

28. How do you rate your family relationships during lock-down?

- Improved
- Worsened
- Unchanged
- I do not know

29. What were your daily life habits during lock-down?

- I prefer to stay in pajamas all day
- I wake up and dress as if I am going out
- I prefer to eat when I feel like it
- I eat at regular times
- I sleep when I feel like it
- I sleep at regular times

30. Did you engage in recreational activities before the COVID-19 emergency?

- Yes
- No

If so, which ones:

- Recreational sport activities
- Artistic activities (music, theater, dance, painting, etc.)
- Recreational activities (volunteering, religious activitties, social activities, etc.)
- Other (specify…...)

Are you continuing these recreational activities?

- Yes
- No

31. Does social distancing and the use of face masks cause problems on you?

- Yes
- No

If so, which ones:

- The face mask does not allow me lip reading
- The face mask reduces the voice volume
- The face mask reduces my olfactory sense, thus compromising the recognition of the surrounding environment
- Social distancing does not allow me to touch people.
- Other (specify ....)

32. Which of your everyday activites were you missing the most during lock-down?
....

33. Are you worried about the risk of SARS-CoV-2 contagion and hospitalization?
- o Not at all
- o To some extent
- o A lot

34. How has your perceived health state changed as compared to before the COVID-19 emergency?
- o Improved
- o Unchanged
- o Worsened

35. Have you experienced sleep disturbances during lock-down?
- o Yes
- o No

If so, did you suffer from it even before?
- o Yes
- o No

36 Are you worried about the future?
- o Not at all
- o To some extent
- o A lot

37. Did you manage to keep contact with friends/family through remote modern technology during lock-down?
- o Yes
- o No

If so, how do you rate your family relationships during lock-down?
- o Improved
- o Unchanged
- o Worsened
- o I do not know

38. Which health protection measure caused the greatest discomfort to you during the COVID-19 emergency?
...

39. What solutions do you suggest to improve the quality of life of disabled individuals during the COVID-19 emergency?
....

Can you please indicate who completed the questionnaire?

- o The disabled person, autonomously
- o The disabled person, with the support of an adult
- o An adult, on behalf of the disabled person
- o Other (specify ....)

THANK YOU FOR YOUR TIME

According to Italian Law 675/1996 and subsequent Legislative Decree 196/2003, all information collected by questionnaires will be used exclusively for scientific research purposes. Data collected by this survey will be anonymized and treated confidentially and can only be shared in aggregate form, so that no individual reference can be identified.
